# Supplementary material for: Facile and Controllable Synthesis of Large-Area Monolayer WS2 Flakes Based on WO3 Precursor Drop-Casted Substrates by Chemical Vapor Deposition
Source: Nanomaterials (Basel). 2019 Apr 9;9(4):578. doi: 10.3390/nano9040578 (PMC6523556; doi:10.3390/nano9040578)
Supplement: Supplementary file 1 [file nanomaterials-09-00578-s001.pdf]

Supplementary material (SM)

Biao Shi [1,†], Daming Zhou[1,†], Shaoxi Fang [1], Khouloud Djebbi [1,2], Shuanglong Feng [1], Hongquan Zhao [1], Chaker Tlili [1,*] and Deqiang Wang [1,2,*]

1 Chongqing Key Lab of Multi-Scale Manufacturing Technology, Chongqing Institute of Green and Intelligent Technology, Chinese Academy of Sciences, Chongqing 400714, China; shibiao@cigit.ac.cn (B.S.); dmzhou@cigit.ac.cn (D.Z.); fangshaoxi@cigit.ac.cn (S.F.); khouloud@cigit.ac.cn (K.D.); fengshuanglong@cigit.ac.cn (S.F.); hqzhao@cigit.ac.cn (H.Z.)

2 University of Chinese Academy of Sciences, Beijing 100049, China

* Correspondence: chakertlili@cigit.ac.cn (C.T.); dqwang@cigit.ac.cn (D.W.)

† These authors contributed equally to this work.

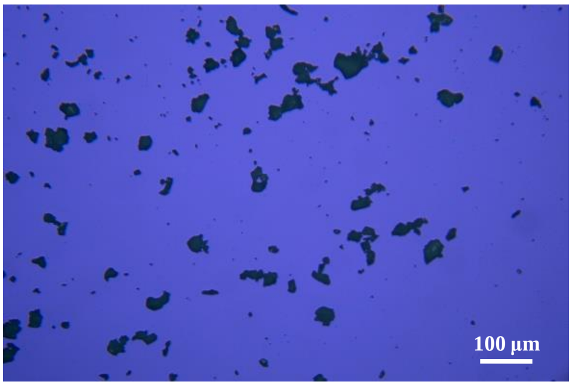

Figure S1. Optical image of powdered WO_3 on SiO_2/Si substrate after the evaporation of ethanol.

Figure S1 shows the typical optical image of powdered WO_3 on SiO_2/Si substrate after the evaporation of ethanol. Although the dimension of WO_3 powders is ranged from a few to dozens of micrometers, relatively uniform distribution of powdered WO_3 can be obtained.

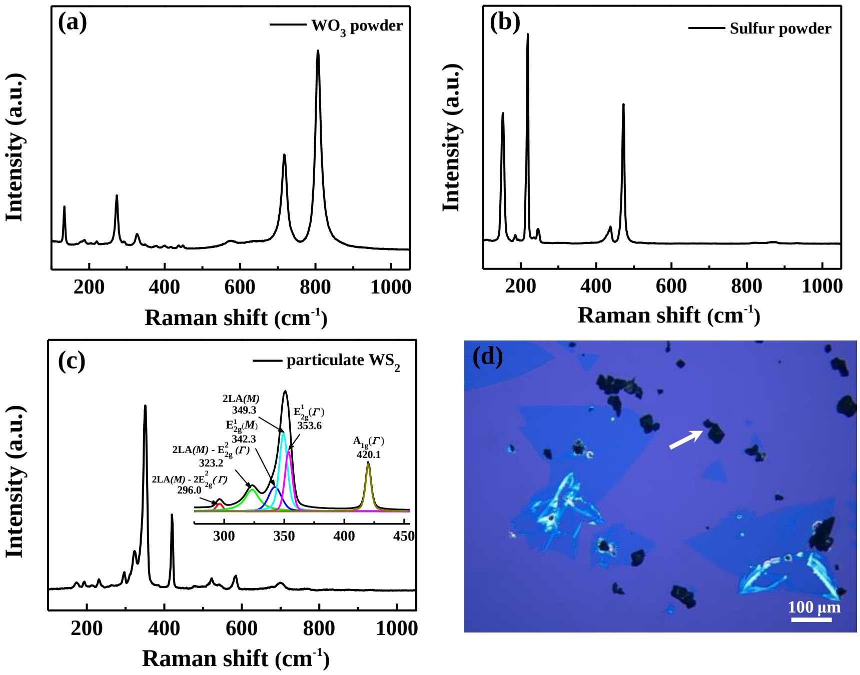

Figure S2. Raman spectra of the WO_3 powder (a), sulfur powder (b) and particulate WS_2 (c) marked with white arrow in Figure.S2 (d). Representative optical image of the as-grown WS_2 sample on the bottom SiO_2/Si substrate that was drop-casted by WO_3-ethanol solution (d).

Figure S2 (a-c) show Raman spectra of the WO_3, sulfur powders and particulate materials grown on the bottom SiO_2/Si substrate. It can be clearly seen that the particulate materials shown in the optical image (d) only exhibit Raman modes related to WS_2, indicating that there are no tungsten oxide and sulfur inclusions in the particulate WS_2.

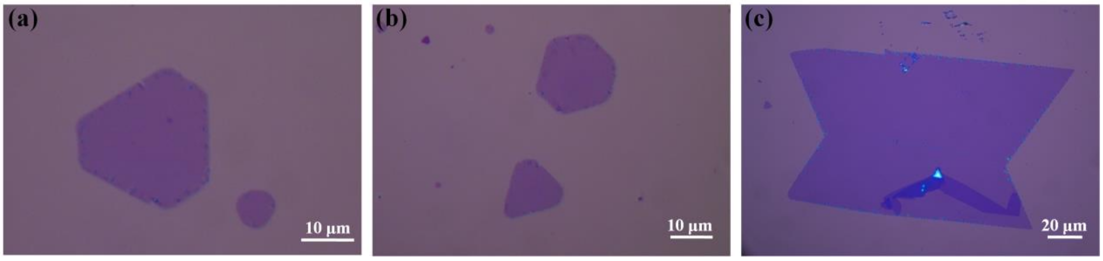

Figure S3. Optical images of as-grown WS_2 flakes on SiO_2/Si substrate with various morphologies: Truncated-triangle (a), Hexagon (b, Butterfly (c).

For WS_2, its crystal structure belongs to P63/mmc space group, and its planar projection shows a perfect hexagonal lattice of sulfur atoms with interleaved tungsten atoms coordinated by sulfur in a trigonal prismatic arrangement [1, 2]. Generally, triangular monolayer WS_2 domains have been commonly observed on the SiO_2/Si substrate. However, under such conditions various morphologies of WS_2 with truncated-triangle, hexagon and butterfly shaped domains can occasionally be identified, as shown in Figure S3.

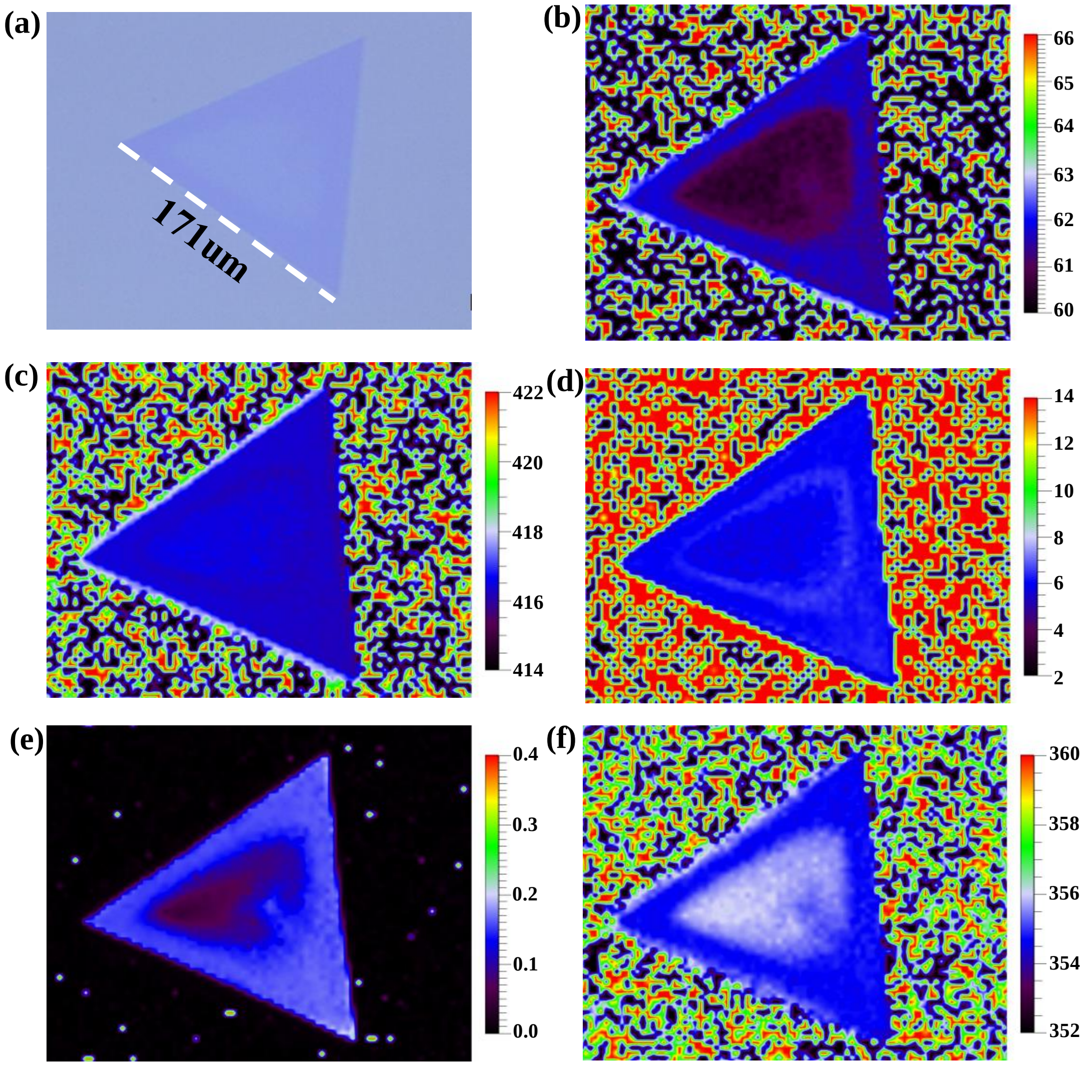

Figure S4. Optical image of the WS_2 flake with edge length of ~ 171 μm on SiO_2/Si substrate (a). Raman mappings of the specific WS_2 sample: frequency difference between $A_{1g}(\Gamma)$ and $E_{2g}^1(\Gamma)$ modes (b); frequency (c), full width of half maximum (FWHM) (d) and normalized intensity (e) of $A_{1g}(\Gamma)$ mode; frequency of $E_{2g}^1(\Gamma)$ mode (f).

The optical image and Raman mapping results displayed in Figure S4 demonstrate that this smaller WS_2 triangle has similar features as the larger one previously observed in Figure 4a and the heterogeneity of FL emission is ascribed to the inhomogeneous tensile strain as well.

References

[1] Schutte, W. J.;Deboer, J. L.; Jellinek, F. CRYSTAL-STRUCTURES OF TUNGSTEN DISULFIDE AND DISELENIDE. Journal of Solid State Chemistry 1987, 70, 207–209.

[2] Elias, A. L.;Perea-Lopez, N.;Castro-Beltran, A.;Berkdemir, A.;Lv, R. T.;Feng, S. M.;Long, A. D.;Hayashi, T.;Kim, Y. A.;Endo, M.;Gutierrez, H. R.;Pradhan, N. R.;Balicas, L.;Houk, T. E. M.;Lopez-Urias, F.;Terrones, H.; Terrones, M. Controlled Synthesis and Transfer of Large-Area WS2 Sheets: From Single Layer to Few Layers. ACS Nano 2013, 7, 5235–5242.
